# Mental health care and pragmatic shared decision making in general practice: an interview study

**DOI:** 10.3399/BJGPO.2024.0111

**Published:** 2025-04-24

**Authors:** Désanne Noordam, Monique Heijmans, Janneke Noordman, Tim olde Hartman, Sandra van Dulmen

**Affiliations:** 1 Nivel (Netherlands Institute for Health Services Research), Utrecht, The Netherlands; 2 Department of Primary and Community Care, Radboud University Medical Center, Radboud Institute for Health Sciences, Nijmegen, The Netherlands

**Keywords:** decision making, shared, mental health, general practitioners

## Abstract

**Background:**

Healthcare providers in general practice are expected to deliver mental health care to patients through shared decision making (SDM). It is unclear whether they perceive their SDM to be affected by challenging circumstances in mental healthcare; for example, how waiting time for therapy plays a role.

**Aim:**

To explore how healthcare providers and patients in general practice engage in SDM, given the challenging circumstances in mental health care.

**Design & setting:**

A qualitative interview study in seven Dutch general practices.

**Method:**

Semi-structured interviews were conducted with GPs (*n* = 9), practice nurses mental health (PNMHs; *n* = 8), and patients who sought mental health care (*n* = 18). The interviews were deductively and inductively thematically analysed.

**Results:**

The participants mainly reported on SDM regarding treatment in secondary mental health care. The PNMHs explained they lack an overview of available treatments and waiting times in facilities. The PNMHs therefore instruct patients to also search for options themselves. Most patients found this approach burdensome, especially those new to mental health care. These patients were said to often express no strong treatment preferences and rely on advice by their healthcare providers. The GPs and PNMHs explained that in such cases, they often adopt a pragmatic approach and, for example, refer indecisive patients to facilities with little waiting times.

**Conclusion:**

The healthcare providers and patients in general practice report that they adapt their approach to SDM in reaction to the circumstances in mental health care. Further exploration of how SDM is implemented and shaped by challenging circumstances across different healthcare settings is needed.

## How it fits in

Healthcare providers in general practice are expected to deliver mental health care to patients through shared decision making (SDM). General factors that influence how they engage in SDM have previously been identified such as the amount of time available. Our research provides in-depth insights into how healthcare providers in general practice engage in SDM with patients who seek mental health care, and how they experience their approach to SDM is shaped by the challenging circumstances in mental health care. These insights may help healthcare providers in general practice to better understand their approach to SDM and tailor it to patients’ individual SDM needs and abilities.

## Introduction

In The Netherlands, a rising number of people are seeking mental health care.^1^ In 2022, 11% of the Dutch population (1.87 million people) was registered in general practice as suffering from one or more mental illnesses.^2^ General practice is often where Dutch patients start their trajectory in mental health care. They visit their GP or practice nurse mental health (PNMH) to receive mental health care in general practice and/or for support to find treatment in secondary mental health care. Generally, treatment in general practice is for patients with mild mental health issues, for example, a mild depression; treatment in secondary mental health care is for patients with more complex or severe mental illnesses, for example, personality disorders.^3^

GPs and PNMHs are expected to deliver mental health care through shared decision making (SDM) and involve their patients in deciding on treatment.^4,5^ Following the commonly adopted model by Elwyn *et al*,^6^ SDM consists of three elements: the healthcare provider (1) acknowledges the patient has options and will help them consider those options; (2) provides information about the options; and (3) stimulates the patient to express their preferences in order to make a shared treatment decision. So far, evidence is inconclusive on whether implementation of SDM improves clinical outcomes in patients who suffer from mental illness.^7^ Nonetheless, patient-related outcomes, such as treatment satisfaction and adherence, do tend to improve when SDM is implemented.^8–10^ Moreover, studies overall show that patients in mental health care wish to be involved in deciding on treatment.^11,12^

Patients who seek secondary mental health care nowadays likely face a significant amount of waiting time before they are accepted into therapy. Mid 2022, around 85 000 Dutch patients were on a waiting list for therapy, with waiting times that ranged from weeks up to months.^13^ Facilities can also decide to temporarily halt their intake of new patients altogether. Partly because of those circumstances, an increasing number of patients turn to general practice for treatment.^14^ Patients also rely on their GP and PNMH to provide temporary care during their waiting time for secondary mental health care. At the same time, Dutch general practices are dealing with limited capacity and staff shortages as well.^15^

Arguably, such circumstances may limit patients in their choice of treatment options and/or their chance to receive timely mental health care. A few studies theorise that restricting treatment options and working with waiting lists for therapy forces patients to consider alternate mental healthcare services.^16,17^ Several other studies conclude that primary care providers consider factors such as accessibility of mental healthcare services in their decision making.^18,19^ Nonetheless, from the literature it remains unclear if and how healthcare providers and patients deliberate and discuss such factors in deciding on treatment.

As healthcare providers in general practice are expected to deliver mental health care to patients through SDM, it is important to gain insight into how they engage in SDM given the challenging circumstances in mental health care. Such insight can help to better understand their process of SDM in its actual healthcare context. Moreover, it can help identify which areas deserve attention in order to facilitate SDM between healthcare providers in general practice and patients who seek mental health care. In our study, we therefore explore how patients and healthcare providers in general practice engage in SDM and how they perceive their SDM is shaped by the challenging circumstances in mental health care.

## Method

### Research design, participants, and recruitment

#### Research design and setting

We used the Consolidated criteria for reporting qualitative research (COREQ) checklist^20^ to report on our research (see Appendix). An explorative, qualitative research design was applied in this study. Semi-structured interviews were conducted in Dutch general practices, with both healthcare providers and patients who sought mental health care.

#### Patient inclusion criteria

Patients were eligible to participate when they had either: depressive symptoms or a diagnosed depression; or a diagnosed personality disorder, for example, borderline personality disorder. In the first place, these two conditions served as inclusion criteria because their prevalence is relatively high in Dutch general practice.^21^ Furthermore, patients with depressive symptoms can likely receive treatment in general practice, as depression care in Dutch general practice is well-established in terms of guidelines^22^ and available eHealth modules. In contrast, mental health care in Dutch general practice for personality disorders is less established; these more complex conditions likely require treatment in secondary mental health care. By focusing on these two patient groups, we expected to gain insight into the SDM process regarding treatment in general practice and in secondary mental health care.

#### Recruitment of healthcare providers and patients

The healthcare providers, that is, the GPs and PNMHs, were recruited via the Academic General Practitioners Network of Radboud University Medical Center (in Dutch ‘Radboud Academisch Huisartsen Netwerk’). Our invitation and information letter were distributed among the 350 Dutch general practices that are part of this network. Twelve general practices responded to our invitation, seven of which agreed to participate.

Patients were recruited in two ways. In the first approach, the GPs and PNMHs who participated selected eligible patients in their general practice. They approached those patients during one of their regular visits and briefly informed them about the focus of our study and participation in the interview. The GPs and PNMHs then handed out our information letter, which contained information about the purpose and procedure of our study and the names, occupations, and contact information of the researchers who would conduct the interviews (MH, DN). Patients who wished to participate had to contact one of the researchers themselves to apply. For the second approach, we recruited patients through two patient organisations: the Depression Association (‘Depressie Vereniging’ in Dutch) and the Borderline Foundation (‘Stichting Borderline’ in Dutch). These organisations disseminated our invitation and information letter in their network and members, that is, patients could contact our researchers to participate. It remains unknown how many patients in total were approached by the healthcare providers and patient organisations and how many patients refused to participate.

### Data collection

#### Interview procedure

The interviews were conducted by two female researchers: one senior (PhD; MH) and one junior (MA, MS; DN). Both researchers were employed in the field of healthcare communication research and had former experience with conducting interviews. The researchers established no further relationship with the participants before the interviews.

The interviews were conducted between December 2021 and February 2022, online via Zoom. Each interview was scheduled to last 1 hour. The patients were interviewed individually, and the healthcare providers individually or in pairs. Each participant signed an informed consent form before their interview. All interviews were audio-recorded and transcribed verbatim. After a participant authorised the transcript, their recording was deleted. The general practices received 500 EUR (approximately 428 GBP) for the efforts of their healthcare providers. Patients were compensated with a 25 EUR (approximately 21 GBP) gift card.

#### Interview content

The interview guides were developed by the complete research team (MH, DN, SvD). For the patient interviews, the aim was to enable participants to naturally share their personal experiences with mental health care. Hence, patients were invited to report on their care trajectory from the onset of their mental illness up to their current situation. During the interviews the researchers paid special attention to patients’ experiences with deciding on treatment. They would pose questions that stimulated the patient to reflect and elaborate on their decision making; for example, ‘How were you involved in the decision making?’ or ‘What were important considerations for you in deciding on treatment?’. The patients were also encouraged to reflect on how the challenging context in mental health care had influenced their decision making, through questions such as ‘In which way did waiting time for therapy play a role in your decision making?’.

The healthcare provider interviews were more structured than the patient interviews, as the GPs and PNMHs had to reflect on topics more generally than personally. The interview guide covered the following themes: mental health care in general practice and secondary mental healthcare; SDM regarding treatment; and the challenging context in mental health care.

### Data analysis

Data analysis and data collection were partly parallel processes; that is, the interview transcripts were analysed during the interview period. This was an iterative process, in which preliminary results served as indicators for whether or not our initial interview guides required revision. Revision is, for instance, necessary when participants address themes that are not covered in the interview guide or when certain themes deserve a different focus.^23^ Ultimately no significant changes were made to our interview guides, as our results appeared to capture the perspectives of both the patients and healthcare providers in a nuanced way.

#### Inductive and deductive thematic analysis

The data analysis consisted of an initial inductive and consecutive deductive thematic analysis. The inductive analysis was performed by the same two researchers who conducted the interviews (MH, DN). To familiarise themselves with the data, the researchers independently coded two transcripts bottom-up, after which they compared and aligned their approaches of assigning codes. As the two researchers independently continued inductively coding the other transcripts, they also started to cluster their codes to identify themes. To do so, they roughly used the themes covered in the interview guides. The researchers met twice to compare and review their initial themes. Afterwards, they extensively discussed all the themes they identified and decided on a final list.

A subsequent deductive thematic analysis was performed on the transcripts, specifically on the subject of SDM. This analysis was guided by the theoretical framework of the ‘observing patient involvement’, that is, the OPTION(5).^24^ This measure is based on the three-talk model of SDM by Elwyn *et al* ^6^ and is originally used to observe SDM in recorded patient–provider interactions. The OPTION(5) consists of five elements, which combined describe the process of SDM. In the deductive thematic analysis, these five elements served as main themes to which codes had to link (see Table 1). The interview transcripts were coded by one researcher (DN) with a background in research on SDM. Findings were repeatedly presented and discussed with the research team (MH, SvD) for critical appraisal until consensus was reached.

**Table 1.** The five elements of shared decision making, according to the OPTION(5) measure,^24^ which served as main themes in the deductive thematic analysis of the interview transcripts

OPTION(5) items	Main theme	Item description from OPTION(5) coding manual
Item 1	‘Confirmation talk’	*’For the health issue being discussed, the clinician draws attention to or confirms that alternate treatment or management options exist or that the need for a decision exists. If the patient rather than the clinician draws attention to the availability of options, the clinician responds by agreeing that the options need deliberation.’*
Item 2	‘Team talk’	*’The clinician reassures the patient or re-affirms that the clinician will support the patient to become informed or deliberate about the options. If the patient states that they have sought or obtained information prior to the encounter, the clinician supports such a deliberation process.’*
Item 3	‘Option talk’	*’The clinician gives information or checks understanding about the options that are considered reasonable (this can include taking no action), to support the patient in comparing alternatives. If the patient requests clarification, the clinician supports the process.’*
Item 4	‘Preference talk’	*’The clinician makes an effort to elicit the patient’s preferences in response to the options that have been described. If the patient declares their preference(s), the clinician is supportive.’*
Item 5	‘Decision talk’	*’The clinician makes an effort to integrate the patient’s elicited preferences as decisions are made. If the patient indicates how best to integrate their preferences as decisions are made, the clinician makes an effort to do so*.’

## Results

Seven general practices located in different parts of The Netherlands participated in our study. In total, nine GPs and eight PNMHs were interviewed. Of the GPs, six were women and three were men, their work experience ranged from 1.5–35 years. All PNMHs were women and had 1–12 years of work experience. In total, 18 patients applied to participate in our study, none dropped out during the research period. Twelve of the patients were female and six were male, their ages ranged from 27–58 years. Eleven patients (had) experienced depression or depressive symptoms, and seven were diagnosed with a personality disorder, mostly borderline. The patients differed in terms of the duration of their mental health problems; some had developed problems 1–2 years ago, while others had lifelong problems. Accordingly, the patients’ treatment histories varied as well; some had recently started their first treatment in general practice, while others had received multiple treatments in secondary mental health care.

### Shared decision making

Most patients who (had) received treatment in general practice were not able to in detail recall how their decision to stay in general practice came about, they mentioned something along the line of ‘I just wanted and got help’. The GPs and PNMHs also pointed out their SDM and treatment of patients in general practice proceeds as usual, they only noticed more and more patients rely on their support during waiting time for treatment. Overall, the patients and healthcare providers mainly reported on their SDM regarding referral to secondary mental health care. They felt that the challenging circumstances in secondary mental health care influenced how they decided on treatment. Hence, the results we present in this section cover only the subject of SDM in relation to treatment in secondary mental health care.

#### Prolonged focus on diagnosing

The GPs and PNMHs explained they do not engage in SDM right from the start; they initially perform diagnostics to assess whether a patient can receive mental health care in general practice or should be referred to secondary mental health care. According to the GPs and PNMHs, they usually deliver mental health care in this order. The health care providers, however, also mentioned they would normally send patients with potentially complex mental health issues through to secondary mental health care for extensive diagnosing. Owing to long waiting time in those facilities, the GPs and PNMHs explained they often refrain from initially doing so. Instead, the healthcare providers indicated they spend more time on diagnosing patients themselves. Several GPs and PNMHs mentioned they could gain assistance from professionals in secondary mental health care; for example, through e-consultations with a psychiatrist. The GPs and PNMHs explained that with the help of those professionals, they aim to reach relatively precise initial diagnoses in order to direct patients towards facilities that can likely accept them into therapy.

#### Incomplete overview of available treatment options and estimates of waiting times

The GPs and PNMHs mentioned that once SDM is initiated, they start off by looking at treatment options together with the patient. According to the healthcare providers, PNMHs are more knowledgeable about treatments and have more time available compared with GPs. Hence, the PNMHs were said to most extensively assist patients in their search for adequate secondary mental health care. Nonetheless, the PNMHs expressed they lack certain information to disclose to their patients, namely a complete overview of available treatment options in secondary mental health care and accurate estimates of waiting times. Some of the PNMHs indicated they would repeatedly contact facilities to inquire about their waiting times. The PNMHs, however, had all ceased to do so, as they considered it a never-ending task.

#### Shifting responsibility to search for treatment towards the patient

The PNMHs explained that they often instruct patients to partly search for treatments in secondary mental health care themselves. According to the PNMHs, they mainly adopted this approach owing to their own lack of overview of treatment options (see paragraph above). The PNMHs mentioned that their approach, however, also enables patients to explore which options appeal to them and match their preferences.

Most of the patients interviewed in this study confirmed that their PNMH (or GP) had instructed them to partly search for treatments themselves. Several patients expressed they found this approach burdensome. These patients explained it was especially difficult for them to actively search for treatments at the time of their first referral, when the whole process was new to them. The patients also struggled with their search at times when they felt overwhelmed by their mental problems (see quote below):

*Patient: ‘*[GP] *did not really know where I could turn to, so she advised me to search the internet for facilities that offered* [type of therapy]*.’*

*Researcher: ‘So* [GP] *placed this responsibility* [to search for options] *with you really?’*

*Patient: ‘Yes. And so I did* [search]*. And there were like 60 or 80 places that offered* [type of therapy]*.’*

*Researcher: ‘How did you feel about the GP instructing you to look for options yourself?’*

*Patient: ‘Well, I felt lost of course, my* [mental] *resilience was like zero. I had never been in contact with mental healthcare, so everything was abracadabra to me really. You have to have quite a bit of perseverance.*’ (Patient 1, depressive symptoms)

#### Patient preferences and the role of waiting time

The GPs and PNMHs noticed that patients vary in how strongly they express treatment preferences. According to the healthcare providers, patients with a newly developed mental health issue and/or no treatment history often express no strong treatment preferences. Most patients confirmed they had no strong treatment preferences at the time of their first referral and relied more on advice by their GP or PNMH. Several GPs and PNMHs indicated they sometimes advise such patients to register for treatment at a secondary mental healthcare facility with relatively short waiting times:

*’With some patients I feel they are better off at a large facility, because those offer more types of treatment. But those places have long waiting lists. Then you might still choose* [to refer the patient to] *a small practice* [with less waiting time] *instead.’* (GP 1)

Most patients interviewed in this study indicated that the amount of waiting time played a role in their decision making, for some to a greater extent than for others:

*’It was either two months* [waiting time] *or half a year, that was an important consideration.* […] *There was one* [facility] *that stood out in terms of short waiting period. So I applied there.’* (Patient 2, personality disorder)

The healthcare providers indicated that patients with a longer treatment history often express stronger treatment preferences and appear more decisive, as they better understand what therapy does and does not work for them. Several patients confirmed they had stronger preferences at the time of their second or third referral. For some patients, this changed how much the waiting time factored into their decision making:

*’With* [facility] *I really urged I wanted to be treated there, because I knew they were specialised in personality disorders. So I just "accepted" that I had to wait. Still though, it was really difficult to wait for so long.’* (Patient 3, personality disorder)

#### Pragmatic approach to decision making

As illustrated in the quotes below, several GPs and PNMHs pointed out they sometimes resort to treatment options that they do not consider the best mental health care. The healthcare providers explained they mainly take such actions when they notice that a patient is indecisive and needs help sooner than they can start therapy in secondary mental health care:

*’Sometimes with depressed patients I prescribe* [antidepressant] *if I know they have to wait three months* [for therapy in secondary mental health care] *and I feel something has to happen right now. I think I am more inclined to take this course of action compared to when a patient can start therapy soon.*’ (GP 2)

*’I have quite some expertise* [from former employment in secondary mental health care] *myself and often patients face much waiting time. In some cases I think, if a patient will likely start a short trajectory* [in secondary mental health care]*, I might as well treat them myself* [in general practice]*.’* (PNMH 1)

## Discussion

### Summary

This study explored how healthcare providers in general practice and patients who seek mental health care engage in SDM, given the challenging circumstances in mental health care. The participants in our study mainly reported on their SDM regarding secondary mental health care. The PNMHs pointed out they lack an overview of treatments and accurate estimates of waiting times in secondary mental health care. The PNMHs explained they therefore instruct patient to also search for options themselves. Most patients found this approach burdensome, especially when they were new to mental health care. These patients were said to often express no strong treatment preferences and rely on advice by their PNMH and GP. The GPs and PNMHs explained that in such cases, they often adopt a pragmatic approach to SDM and, for example, refer indecisive patients to facilities with little waiting time.

### Strengths and limitations

While SDM has been studied in various settings, our research is one of few to very concretely consider and report on SDM in its actual healthcare context. We included the perspectives of both patients and healthcare providers in general practice to identify how they perceive their SDM is shaped by the challenging circumstances in mental health care. These perspectives combined resulted in rich findings. Nonetheless, it should be noted that our findings pertain to the specific circumstances in Dutch general practice and mental health care. The general practice workforce in other countries may, for example, not include staff members with a role similar to that of PNMH. Hence, application of our findings to different mental healthcare settings across other countries may be limited.

### Comparison with existing literature

The GPs and PNMHs in our study mentioned they spend more time on diagnosing patients in general practice before they refer to secondary mental health care. Their objective for this was not to better understand if their referrals are justified, but to increase the chance that their referrals are successful, in the sense that patients can be admitted into therapy and are not needlessly placed on a waiting list. This required the GPs and PNMHs to establish relatively precise preliminary diagnoses. While clarification of a patient’s (mental) problem is a prerequisite to initiate the process of SDM,^25^ a prolonged focus on diagnosing may limit time that is left for SDM. Turning to literature, many studies show that healthcare providers perceive a lack of time as the main barrier to engaging in SDM.^26,27^ In general practice time is scarce as well, and precious time that could be spent on SDM is now necessarily spent on establishing diagnoses.

Second, we found that once SDM is initiated, the PNMHs felt unable to fully inform patients about options in secondary mental health care. The PNMHs mainly lacked an overview of available treatments and estimates of waiting times. Former research shows that healthcare providers often report a lack of informational resources as a barrier to engage in SDM.^28^ The PNMHs in our study made clear they had to put in disproportionate effort to obtain the information they lacked and keep it updated. The PNMHs therefore adopted a pragmatic approach of informing patients, whereby they instruct patients to partly search for treatments themselves. The PNMHs did not apply this approach solely out of practical consideration; according to the PNMHs, it also enables patients to explore which treatments, therapists, or practices in secondary mental health care appeal to them. In that sense, the PNMHs’ approach promotes elicitations of patient preferences, an imperative part of SDM that is often overlooked by healthcare providers.^29,30^

While their approach made informing patients more workable for the PNMHs, it did not resonate well with the SDM needs and abilities of all patients in our study. Multiple patients explained it felt burdensome having to actively search for options in secondary mental health care themselves. Similar findings are reported in research by Grung *et al*.^12^ Previous research also shows that patients’ ability to actively participate in SDM fluctuates according to the severity of their mental problem and across treatment stages.^31^ In our study, patients who were new to mental health care and the process of referral appeared to struggle the most, as they were unable to draw from former experience. Accordingly, these patients were said to often express no strong treatment preferences and rely on advice by their GP and PNMH. With indecisive patients, the GPs and PNMHs appeared to shift towards more pragmatic decision making. They would, for instance, refer those patients to facilities with little waiting time. In some cases, the GPs and PNMHs decided to keep patients in general practice even though secondary mental health care could be a fit. They, for instance, did so with patients who required short-term and a relatively light form of therapy. The PNMHs who were formerly employed in secondary mental health care felt confident they might as well treat those patients in general practice and spare them waiting time. Through such trade-off decisions, patients may not necessarily end up in secondary mental health care that is not tailored to their needs, but they might. In any case, the circumstances in mental health care appear to challenge patients, GPs, and PNMHs in letting their SDM be solely guided by the aim to find adequate mental health care.

### Implications for research and practice

Our research underlines the importance of considering the context-sensitive nature of SDM. We recommend future studies to further explore how SDM is implemented and shaped by (challenging) circumstances across different healthcare settings. Such knowledge may enable both policymakers and healthcare providers to implement SDM in a way that is more tailored to their specific circumstances, instead of adopting a ‘one-size-fits-all’ approach. Healthcare providers in Dutch general practice that take notice of our study may apply our findings to their SDM with patients who seek mental health care. In particular patients new to mental health care may benefit from a different approach to SDM than is currently adopted. The healthcare providers may, for instance, involve informal caregivers in the decision-making process to help the patient search for treatment that is tailored to them personally.

## Data

The dataset relied on in this article is available from the corresponding author on reasonable request.
